# In vitro inhibition of human cytochrome P450 enzymes by the novel atypical antipsychotic drug asenapine: a prediction of possible drug–drug interactions

**DOI:** 10.1007/s43440-020-00089-z

**Published:** 2020-03-26

**Authors:** Jacek Wójcikowski, Przemysław J. Danek, Agnieszka Basińska-Ziobroń, Renata Pukło, Władysława A. Daniel

**Affiliations:** Department of Pharmacokinetics and Drug Metabolism, Maj Institute of Pharmacology, Polish Academy of Sciences, Smętna 12, 31-343 Kraków, Poland

**Keywords:** Asenapine, Cytochrome P450, Inhibition, Human liver microsomes, cDNA-expressed CYP enzymes

## Abstract

**Background:**

Inhibition of cytochrome P450 (CYP) enzymes is the most common cause of harmful drug–drug interactions. The present study aimed at examining the inhibitory effect of the novel antipsychotic drug asenapine on the main CYP enzymes in human liver.

**Methods:**

The experiments were performed in vitro using pooled human liver microsomes and the human cDNA-expressed CYP enzymes: CYP1A2, CYP2C9, CYP2C19, CYP2D6, and CYP3A4 (Supersomes). Activities of CYP enzymes were determined using the CYP-specific reactions: caffeine 3-*N*-demethylation (CYP1A2), diclofenac 4′-hydroxylation (CYP2C9), perazine *N*-demethylation (CYP2C19), bufuralol 1′-hydroxylation (CYP2D6), and testosterone 6β-hydroxylation (CYP3A4). The rates of the CYP-specific reactions were assessed in the absence and presence of asenapine using HPLC.

**Results:**

The obtained results showed that both in human liver microsomes and Supersomes asenapine potently and to a similar degree inhibited the activity of CYP1A2 via a mixed mechanism (*K*_*i*_ = 3.2 μM in liver microsomes and Supersomes) and CYP2D6 via a competitive mechanism (*K*_*i*_ = 1.75 and 1.89 μM in microsomes and Supersomes, respectively). Moreover, asenapine attenuated the CYP3A4 activity via a non-competitive mechanism (*K*_*i*_ = 31.3 and 27.3 μM in microsomes and Supersomes, respectively). In contrast, asenapine did not affect the activity of CYP2C9 or CYP2C19.

**Conclusion:**

The potent inhibition of CYP1A2 and CYP2D6 by asenapine, demonstrated in vitro, will most probably be observed also in vivo, since the calculated *K*_*i*_ values are close to the presumed concentration range for asenapine in the liver in vivo. Therefore, pharmacokinetic interactions involving asenapine and CYP2D6 or CYP1A2 substrates are likely to occur during their co-administration to patients.

## Introduction

Cytochrome P450 (CYP) enzymes are members of a superfamily of heme-containing monooxygenases catalyzing the metabolism of endogenous substances (e.g., steroids, monoaminergic neurotransmitters, and arachidonic acid) and the majority of clinically important drugs, including psychotropics. CYP1A2, CYP2C9, CYP2C19, CYP2D6, and CYP3A4, constitute 13, 20−30, 4, 1.5, and 30−50% of the total CYP protein in human liver, respectively, and are involved in the metabolism of approximately 90% of all marketed drugs [1–4]. They are pivotal CYP isoforms in the evaluation of CYP-mediated drug–drug interactions.

The catalytic activity of cytochromes P450 includes most often C-oxidation reactions (hydroxylation, epoxidation, and peroxidation), N- and S-oxidation, as well as oxidative O-, S-, or N-dealkylation, and other types of reactions. These reactions are engaged in the first phase of xenobiotic (drug) metabolism and in the biosynthesis and metabolism of endogenous compounds. The primary goal of the first-phase metabolism of xenobiotics by cytochrome P450 is their biotransformation into an inactive form with increased solubility in water to facilitate the deactivation and excretion of xenobiotics from the body [5]. However, metabolism may sometimes result in the formation of active metabolites that have pharmacological activities similar to those of the parent compound or with entirely different biological properties, including the ability to alter the metabolism of other chemical compounds. That situation applies to many chemical carcinogens, while the increased biological activity is desirable for pro-drugs [6].

Asenapine is a new atypical antipsychotic drug that has been developed as a structural modification of the antidepressant drug mianserin. Chemically, asenapine is categorized within the family of dibenzoxepinopyrrolidines [7]. The drug is currently approved for the acute and maintenance treatment of schizophrenia as well as for acute treatment of manic or mixed episodes associated with bipolar I disorder with or without psychotic features as monotherapy or adjunctive medication along with lithium or valproate. Asenapine has a unique human receptor-binding profile characterized by an antagonistic action at serotonergic (5-HT_2_, 5-HT_5–7_), adrenergic (*α*_1_ and *α*_2_), dopaminergic (*D*_1–4_), and histaminergic H_1_ receptors, but produces no action on the β-adrenergic or muscarinic receptors [8, 9]. Due to its pharmacological profile, asenapine is effective not only against positive (psychotic) symptoms by affecting dopaminergic receptors, but it is also efficient against negative symptoms of schizophrenia improving mood and cognition (via 5-HT_1A_, HT_2C_, 5-HT_6_, and 5-HT_7_), and simultaneously protecting against extrapyramidal symptoms (via 5-HT_2A_) [9, 10]. Asenapine is metabolized to 38 different metabolites, none of which has any significant functional activity at receptors. Asenapine appears to be metabolized via four primary metabolic pathways to *N*-desmethylasenapine, 11-hydroxy-asenapine, asenapine *N*-oxide, and asenapine *N*-glucuronide. With the exception of the glucuronide, the primary metabolites are all further extensively metabolized. The primary mechanism of asenapine metabolism involves glucuronidation through UDP glucuronosyltransferase 1A4 (UGT1A4), producing asenapine-*N*-glucuronide. The other major metabolite of asenapine is produced through demethylation, resulting in *N*-desmethylasenapine, primarily via CYP1A2, with only minor contributions of CYP3A4 and CYP2D6. The estimated terminal half-life (*t*_1/2_) in adults is approximately 24 h. Asenapine is rapidly distributed, has a large volume of distribution, and is highly bound to plasma proteins (95%). Elimination of asenapine and its metabolites occurs approximately equally via hepatic and renal routes [7, 11, 12].

Direct inhibition of CYP enzymes is the most common cause of harmful drug–drug interactions. Inhibition of drug metabolism can lead to a decreased elimination and increased bioavailability (a decreased first-pass effect) of the parent compound. If a drug is metabolized mainly via a single metabolic pathway, its inhibition may result in an increased steady-state concentration and accumulation ratio, and non-linear kinetics as a consequence of enzyme saturation. In the case of pro-drugs, enzyme inhibition may result in a decrease in the amount of the active drug form [4, 13].

Previous in vitro metabolic studies have shown that typical neuroleptics (phenothiazines) are potent inhibitors of human CYP2D6 (thioridazine, chlorpromazine, levomepromazine, and perhenazine) and CYP1A2 (perazine), as well as week inhibitors of CYP1A2, CYP2C9, CYP2C19, or CYP3A4. In comparison, the older atypical antipsychotics, like clozapine, olanzapine, risperidone, and zipasidone, moderately diminish the activity of CYP2D6, and weakly inhibit that of CYP3A4, CY2C9, and CYP2C19 in human liver [14–19].

Although there are some experimental data showing a possibility of inhibition of CYP isoforms by typical and older atypical neuroleptics, the inhibition studies of human CYP enzymes by the novel antipsychotic drug asenapine have not been presented, so far. Therefore, this work aimed at examining the inhibitory effect of asenapine on the main human liver CYP enzymes: CYP1A2, CYP2C9, CYP2C19, CYP2D6, and CYP3A4.

## Materials and methods

### Drugs and chemicals

Asenapine, caffeine, 3-*N*-desmethyl caffeine (paraxanthine), diclofenac, 4′-hydroxydiclofenac, bufuralol, 1′-hydroxybufuralol, NADP, NADPH, glucose-6-phosphate, glucose-6-phosphate-dehydrogenase, MgCl_2_, KCl, ZnSO_4_, Trizma base, and ethylenediaminetetraacetic acid (EDTA) were purchased from Sigma (St. Louis, USA). Testosterone and 6β-testosterone were from Steraloids (Newport, USA). All the organic solvents with HPLC purity were supplied by Merck (Darmstadt, Germany). Pooled human liver microsomes and microsomes from baculovirus-infected insect cells expressing human CYP1A2, CYP2C9, CYP2C19, CYP2D6, and CYP3A4 (Supersomes) were provided by Corning (Woburn, USA).

### Determination of CYP enzyme activities

#### CYP1A2 activity assay

The activity of CYP1A2 was investigated by measuring the rate of a CYP1A2-specific reaction, i.e., 3-*N*-demethylation of caffeine, as described previously [20]. Briefly, incubations were carried out in a system containing pooled human liver microsomes (ca. 0.5 mg of protein/ml), a phosphate buffer (0.15 M, pH 7.4), and NADPH (1 mM). The rate of caffeine 3-*N*-demethylation was assessed at the substrate concentrations of 200, 400, and 800 μM, in the absence and presence of asenapine, added in vitro (asenapine concentrations: 0.01, 0.05, 0.1, 0.5, 1, 5, and 10 μM). The final incubation volume was 0.5 ml. After a 50-min incubation, the reaction was terminated by adding 700 μl of a 2% ZnSO_4_ and 50 μl of 2 M HCl. In the case of microsomes from baculovirus-infected insect cells expressing CYP1A2 (Supersomes 1A2), the effect of asenapine on the CYP1A2 activity was studied under experimental conditions similar to those described for human liver microsomes, except for the fact that the final concentration of CYP1A2 was 50 pmol/ml and the incubation time was 30 min. Concentrations of caffeine and its metabolite 3-*N*-desmethyl caffeine (paraxanthine), produced in liver microsomes or Supersomes 1A2, were assessed using the HPLC method with UV detection, as described previously [20].

### CY2C9 activity assay

The activity of CYP2C9 was examined by measuring the rate of a CYP2C9-specific reaction, i.e., 4′-hydroxylation of diclofenac, as described previously [19]. Briefly, incubations were carried out in a system containing pooled human liver microsomes (ca. 0.5 mg of protein/ml), a Tris/KCl buffer (50 mM, pH = 7.4), MgCl_2_ (3.0 mM), EDTA (1 mM), NADP (1.0 mM), glucose 6-phosphate (5 mM), and glucose-6-phosphate-dehydrogenase (1.7 U in 1 ml). The rate of diclofenac 4′-hydroxylation was assessed at the substrate concentrations of 5, 10, and 25 μM, in the absence and presence of asenapine, added in vitro (asenapine concentrations: 0.01, 0.05, 0.1, 0.5, 1, 5, and 10 μM). The final incubation volume was 0.5 ml. After a 30-min incubation, the reaction was stopped by adding 100 µl of acetonitrile. The effect of asenapine on the CYP2C9 activity in microsomes from baculovirus-infected insect cells expressing CYP2C9 (Supersomes 2C9) was studied under experimental conditions similar to those described for human liver microsomes. The final concentration of CYP2C9 was 50 pmol/ml. Concentrations of diclofenac and its metabolite 4′-hydroxydiclofenac, formed in liver microsomes or Supersomes 2C9, were assessed by the HPLC method with UV detection, as described previously [21].

#### CY2C19 activity assay

The activity of CYP2C19 was estimated by measuring the rate of a CYP2C19-specific reaction, i.e., *N*-demethylation of perazine, as described previously [22]. Briefly, incubations were carried out in a system containing pooled human liver microsomes (ca. 0.5 mg of protein/ml), a Tris/KCl buffer (50 mM, pH = 7.4), MgCl_2_ (3.0 mM), EDTA (1 mM), NADP (1.0 mM), glucose 6-phosphate (5 mM), and glucose-6-phosphate-dehydrogenase (1.7 U in 1 ml). The rate of perazine *N*-demethylation was measured at the substrate concentrations of 50, 100, and 200 μM, in the absence and presence of asenapine, added in vitro (asenapine concentrations: 0.01, 0.05, 0.1, 0.5, 1, 5, and 10 μM). The final incubation volume was 0.5 ml. After a 20-min incubation (liver microsomes), the reaction was stopped by adding 100 µl of methanol. The effect of asenapine on the CYP2C19 activity in microsomes from baculovirus-infected insect cells expressing CYP2C19 (Supersomes 2C19) was studied under experimental conditions similar to those described for human liver microsomes. The final concentration of CYP2C19 was 50 pmol/ml and the incubation time was 30 min. Concentrations of perazine and its metabolite *N*-desmethylperazine, produced in liver microsomes or Supersomes 2C19, were assessed by the HPLC method with UV detection, as described previously [22].

#### CY2D6 activity assay

The activity of CYP2D6 was evaluated on the basis of the rate of a CYP2D6-specific reaction, i.e., 1′-hydroxylation of bufuralol, as described previously [23]. Briefly, incubations were carried out in a system containing pooled human liver microsomes (ca. 0.5 mg of protein/ml), a potassium phosphate buffer (0.1 M, pH = 7.4), MgCl_2_ (3.3 mM), NADP (1.3 mM), glucose 6-phosphate (3.3 mM), and glucose-6-phosphate-dehydrogenase (1.0 U in 1 ml). The rate of bufuralol 1′-hydroxylation was assessed at the substrate concentrations of 10, 25, and 50 μM, in the absence and presence of asenapine, added in vitro (neuroleptic concentrations: 0.01, 0.05, 0.1, 0.5, 1, 5, and 10 μM). The final incubation volume was 0.5 ml. After a 30-min incubation, the reaction was stopped by adding 30 µl of a 70% perchloric acid. The effect of asenapine on the CYP2D6 activity in microsomes from baculovirus-infected insect cells expressing CYP2D6 (Supersomes 2D6) was studied under experimental conditions similar to those described for human liver microsomes. The final concentration of CYP2D6 was 50 pmol/ml. Concentrations of bufuralol and its metabolite 4′-hydroxybufuralol in liver microsomes or Supersomes 2D6 were assessed by the HPLC method with fluorimetric detection, as described previously [23].

#### CY3A4 activity assay

The activity of CYP3A4 was studied by measuring the rate of 6β-hydroxylation of testosterone, a CYP3A4-specific reaction, as described previously [24]. Briefly, incubation was carried out in a system containing pooled human liver microsomes (ca. 0.5 mg of protein/ml), a Tris/KCl buffer (50 mM, pH = 7.4), MgCl_2_ (3.0 mM), EDTA (1 mM), NADP (1.0 mM), glucose 6-phosphate (5 mM), and glucose-6-phosphate-dehydrogenase (1.7 U in 1 ml). The rate of testosterone 6β-hydroxylation was assessed at the substrate concentrations of 50, 100, and 200 μM, in the absence and presence of asenapine, added in vitro (asenapine concentrations: 0.01, 0.05, 0.1, 0.5, 1, 5, and 10 μM). The final incubation volume was 0.5 ml. After a 20-min incubation, the reaction was stopped by adding 200 µl of methanol. The effect of asenapine on the CYP3A4 activity in microsomes from baculovirus-infected insect cells expressing CYP3A4 (Supersomes 3A4) was studied under experimental conditions similar to those described for human liver microsomes. The final concentration of CYP3A4 was 50 pmol/ml and the incubation time was 30 min. Concentrations of testosterone and its metabolite 6β-hydroxytestosterone, formed in liver microsomes or Supersomes 3A4, were assessed by the HPLC method with UV detection, as described previously [24].

### Determination of kinetic parameters, *K*_*i*_ and IC_50_ values, and the mechanism of inhibition

Kinetic parameters (*K*_*m*_, *V*_max_,* K*_*i*_) describing the metabolism of CYP-specific substrates in liver microsomes and Supersomes were obtained using the Michaelis–Menten approach and a non-linear regression analysis (Program Sigma Plot 12.3 Enzyme Kinetics). The inhibitory effects of asenapine on CYP enzymes are presented graphically as Dixon’s plots (1/V against I) indicating *K*_*i*_ values, and Lineweaver–Burk’s plots (1/V against 1/S) showing the mechanism of inhibition (competitive inhibition increases the *K*_*m*_ value, not affecting the *V*_max_ value; non-competitive inhibition decreases the *V*_max_ value, not affecting the *K*_*m*_ value; mixed inhibition entails respective changes in both the *K*_*m*_ and *V*_max_ values).

The IC_50_ values (asenapine concentrations required for 50% inhibition of particular CYP enzymes in vitro) were determined by plotting relative CYP activities vs. the logarithm of asenapine concentrations, using GraphPad Prism 7.0 (GraphPad Prism Software Inc., CA, USA).

## Results

**Fig. 1:**
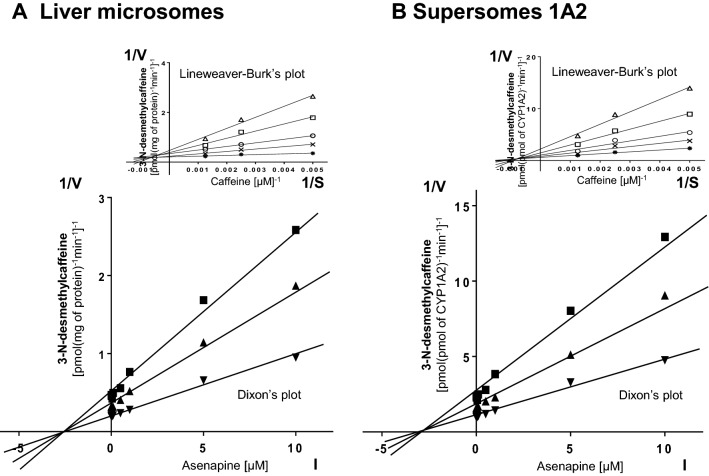
The influence of asenapine on the activity of CYP1A2 measured as a rate of caffeine 3-*N*-demethylation. **a** Human liver microsomes (*K*_*m*_ = 709 µM, *V*_max_ = 9.67 pmol/mg protein/min). **b** Human cDNA-expressed CYP1A2 (Supersomes CYP1A2) (*K*_*m*_ = 705 µM, *V*_max_ = 1.92 pmol/pmol CYP/min). Each point represents the mean value of two independent analyses. *V* velocity of the reaction, *I* the concentration of the inhibitor (asenapine), *S* the concentration of the substrate (caffeine). The *K*_*i*_ values and mechanisms of inhibition are shown in Table 1. Dixon’s plots (the main plots): the caffeine concentration of 200 µM (■), 400 µM (▲), and 800 µM (▼). Lineweaver–Burk’s plots (inserts): control—no asenapine (✱); the asenapine concentration of 0.5 µM (**x**), 1 µM (**○**), 5 µM (**□**), and 10 µM (**△**)

**Fig. 2:**
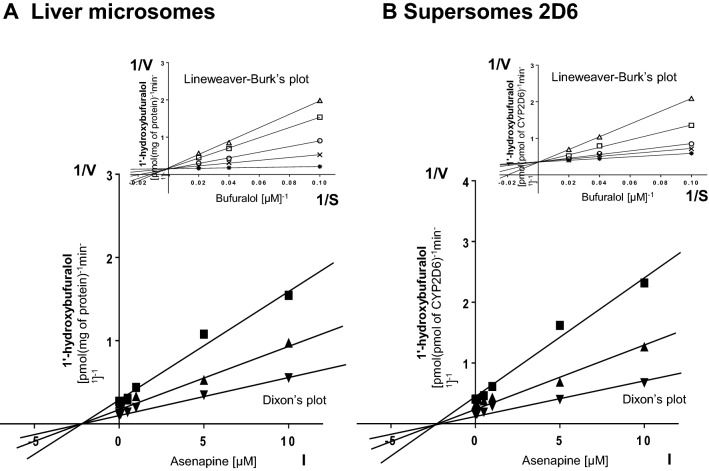
The influence of asenapine on the activity of CYP2D6 measured as a rate of bufuralol 1′-hydroxylation. **a** Human liver microsomes (*K*_*m*_ = 5.1 µM, *V*_max_ = 5.59 pmol/mg protein/min). **B** Human cDNA-expressed CYP2D6 (Supersomes CYP2D6) (*K*_*m*_ = 6.6 µM, *V*_max_ = 4.1 pmol/pmol CYP/min). Each point represents the mean value of two independent analyses. *V* velocity of the reaction, *I* the concentration of the inhibitor (asenapine), *S* the concentration of the substrate (bufuralol). The *K*_*i*_ values and mechanisms of inhibition are shown in Table 1. Dixon’s plots (the main plots): the bufuralol concentration of 10 µM (■), 25 µM (▲), and 50 µM (▼). Lineweaver–Burk’s plots (inserts): control—no asenapine (✱); the asenapine concentration of 0.5 µM (**x**), 1 µM (**○**), 5 µM (**□**), and 10 µM (**△**)

**Fig. 3:**
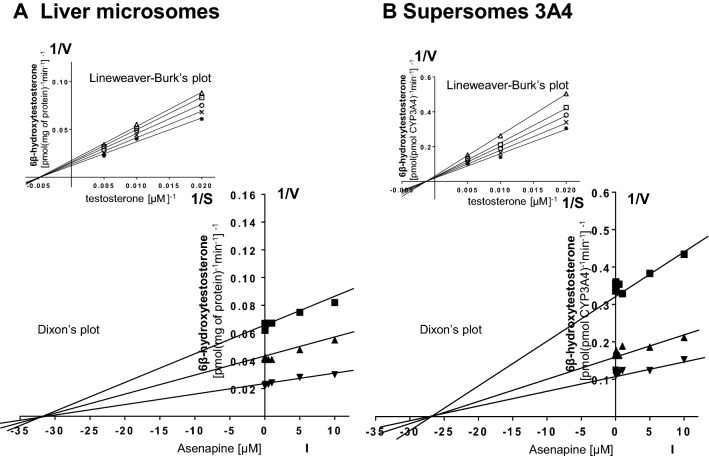
The influence of asenapine on the activity of CYP3A4 measured as a rate of testosterone 6β-hydroxylation. **a** Human liver microsomes (*K*_*m*_ = 283 µM, *V*_max_ = 72.9 pmol/mg protein/min). **b** Human cDNA-expressed CYP3A4 (Supersomes CYP3A4) (*K*_*m*_ = 428 µM, *V*_max_ = 42.9 pmol/pmol CYP/min). Each point represents the mean value of two independent analyses. *V* velocity of the reaction, *I* the concentration of the inhibitor (asenapine), *S* the concentration of the substrate (testosterone). The *K*_*i*_ values and mechanisms of inhibition are shown in Table 1. Dixon’s plots (the main plots): the testosterone concentration of 50 µM (■), 100 µM (▲), and 200 µM (▼). Lineweaver–Burk’s plots (inserts): control—no asenapine (✱); the asenapine concentration of 0.5 µM (**x**), 1 µM (**○**), 5 µM (**□**), and 10 µM (**△**)

Dixon’s plots of the metabolism of CYP-specific substrates, carried out in human liver microsomes and Supersomes CYP1A2, CYP2D6, and CYP3A4, in the absence or presence of asenapine, demonstrated that the examined neuroleptic exerted a significant inhibitory effect on CYP1A2, CYP2D6, and CYP3A4; however, its potency in relation to specific CYP isoenzymes was diverse (main plots in Figs. 1a, b; 2a, b; 3a, b). Both in human liver microsomes and Supersomes asenapine potently and, to a similar degree, inhibited the activity of CYP1A2 (*K*_*i*_ = 3.2 μM in liver microsomes and Supersomes) and CYP2D6 (*K*_*i*_ = 1.75 and 1.89 μM in liver microsomes and Supersomes, respectively). Moreover, asenapine attenuated the CYP3A4 activity (*K*_*i*_ = 31.3 and 27.3 μM in liver microsomes and Supersomes, respectively).

Lineweaver–Burk’s plots indicated that both in human liver microsomes and Supersomes asenapine inhibited the activity of CYP1A2 via a mixed mechanism, CYP2D6 via a competitive mechanism, and that of CYP3A4 via a non-competitive mechanism (inserts in Figs. 1a, b; 2a, b; 3a, b). The *K*_*i*_ values and mechanisms of inhibition of CYP1A2, CYP2D6, and CYP3A4 activities by asenapine are summarized in Table 1.

**Table 1:** The ability of asenapine to inhibit CYP1A2, CYP2D6 and CYP3A4 activities in vitro in human liver microsomes and human cDNA-expressed CYP enzymes (Supersomes)

Inhibitor	Inhibition of CYP-specific reactions by asenapine *K*_*i*_ (μM) and type of inhibition					
	Caffeine 3-*N*-demethylation (CYP1A2)		Bufuralol 1′-hydroxylation (CYP2D6)		Testosterone 6β-hydroxylation (CYP3A4)	
Asenapine	Liver microsomes	Supersomes CYP1A2	Liver microsomes	Supersomes CYP2D6	Liver microsomes	Supersomes CYP3A4
	3.2 (mixed)	3.2 (mixed)	1.75 (competitive)	1.89 (competitive)	31.3 (non-competitive)	27.3 (non-competitive)

The presented inhibition constants (*K*_*i*_) for the inhibition of particular CYP-specific reactions by asenapine were obtained using a non-linear regression analysis (Program Sigma Plot 12.3; Enzyme Kinetics) and are shown graphically in Figs. 1a, b; 2a, b and 3a, b (Dixon’s plots). The mechanisms of inhibition were estimated on the basis of changes in the *K*_*m*_ and/or *V*_max_ values of the tested inhibitor (asenapine) and are shown graphically in the inserts of Figs. 1a, b; 2a, b and 3a, b (Lineweaver–Burk’s plots)

In addition, the IC_50_ values obtained for the asenapine inhibition of cytochrome P450 enzyme activities in vitro in human liver microsomes and human cDNA-expressed CYP enzymes (Supersomes) are shown in Table 2. The obtained IC_50_ values confirmed the ability of asenapine to inhibit the CYP1A2, CYP2D6, and CYP3A4 activities.

**Table 2:** The IC_50_ values obtained for the asenapine inhibition of cytochrome P450 enzyme activities in vitro in human liver microsomes and human cDNA-expressed CYP enzymes (Supersomes)

Enzyme	Marker reaction	Asenapine − IC_50_ (µM)	
		Liver microsomes	Supersomes
CYP1A2	Caffeine 3-*N*-demethylation	0.697	0.751
CYP2C9	Diclofenac 4′-hydroxylation	No inhibition > 1000	No inhibition > 1000
CYP2C19	Perazine *N*-demethylation	No inhibition > 1000	No inhibition > 1000
CYP2D6	Bufuralol 1′-hydroxylation	0.391	0.398
CYP3A4	Testosterone 6β-hydroxylation	6.123	8.682

The IC_50_ values were estimated for the following substrate concentrations: caffeine 400 µM, diclofenac 10 µM, perazine 100 µM, bufuralol 50 µM, and testosterone 100 µM

**Fig. 4:**
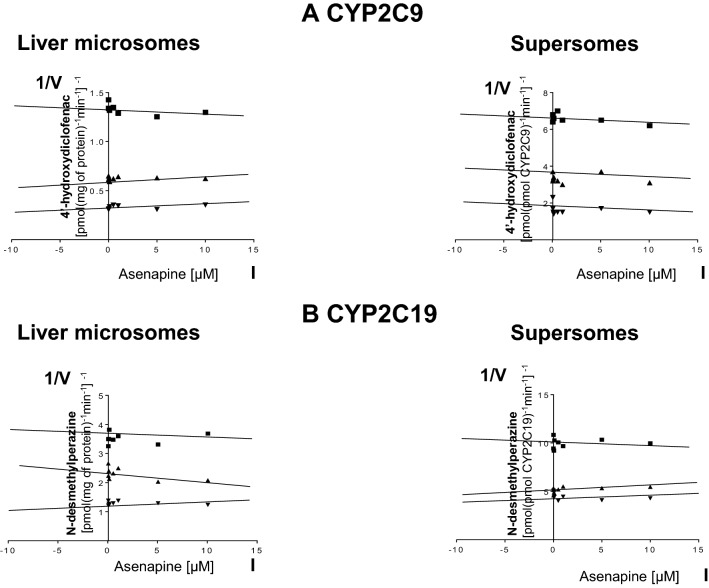
The influence of asenapine on the activity of CYP2C9 (**a**) measured as a rate of diclofenac 4′-hydroxylation and CYP2C19 (**b**) measured as a rate of perazine *N*-demethylation. The enzyme activity was estimated in human liver microsomes (*K*_*m*_ = 136.8 µM, *V*_max_ = 21.02 nmol/mg protein/min for diclofenac 4′-hydroxylation; *K*_*m*_ = 199.9 µM; *V*_max_ = 1.58 nmol/mg protein/min for perazine *N*-demethylation) and Supersomes CYP2C9 and CYP2C19 (*K*_*m*_ = 131.9 µM, *V*_max_ = 213.6 pmol/pmol CYP/min for diclofenac 4′-hydroxylation; *K*_*m*_ = 162.3 µM, *V*_max_ = 39.4 pmol/pmol CYP/min for perazine *N*-demethylation). Each point represents the mean value of two independent analyses. *V* velocity of the reaction, *I* the concentration of the inhibitor (asenapine), *S* the concentration of the substrate (diclofenac or perazine). Dixon’s plots: **a** the diclofenac concentration of 5 µM (■), 10 µM (▲), and 25 µM (▼); **b** the perazine concentration of 50 µM (■), 100 µM (▲), and 200 µM (▼)

On the other hand, asenapine did not affect the activity of CYP2C9 or CYP2C19, neither in liver microsomes, nor in Supersomes (Fig. 4).

## Discussion

When cytochrome P450 is inhibited, the pharmacokinetics of a drug which is metabolized by this enzyme will change, thereby increasing the drug efficacy or causing drug toxicity. As mentioned in the introduction, asenapine is mainly metabolized by CYP1A2, with a small contribution of CYP3A4 and CYP2D6 [7, 11, 12]. Therefore, the use of strong inhibitors of these enzymes may cause drug–drug interactions. Furafylline, quinidine, and ketaconazole are potent inhibitors of CYP enzymes (1A2, 2D6, and 3A4, respectively). When these inhibitors are co-administered with asenapine, the metabolism of the neuroleptic may be inhibited. Then, the plasma concentration of asenapine may increase, and adverse drug–drug interaction might occur. Asenapine is not metabolized by CYP2C9 or CYP2C19. Therefore, the combination of asenapine with selective inhibitors for these enzymes (e.g., sulfaphenazole for CYP2C9 or (–)-*N*-3-benzyl-phenobarbital for CYP2C19) will not affect the metabolism of asenapine.

Another question that arises in drug development is whether a new drug may inhibit the metabolism of other concurrently administered drugs. One approach to answer this question is to examine whether the new drug may inhibit the metabolism of known specific substrates of cytochrome P450 enzymes in vitro. The obtained results demonstrated that asenapine exerted significant inhibitory effects on CYP1A2, CYP2D6, and CYP3A4 activities measured as the rates of caffeine 3-*N*-demethylation, bufuralol 1′-hydroxylation, and testosterone 6β-hydroxylation, respectively. However, its potency in affecting specific CYP enzymes was diverse. Asenapine potently inhibited CYP1A2 and CYP2D6 via a mixed or competitive mechanism (respectively), and weakly diminished the activity of CYP3A4 via a non-competitive mechanism. On the other hand, asenapine did not affect the activities of CYP2C9 and CYP2C19. The results obtained in the two in vitro experimental models are coherent, which strengthen the conclusions drawn from this study. The potent inhibition of CYP1A2 by asenapine displays *K*_*i*_ value which is situated in the range of the *K*_*i*_ values observed for such potent CYP1A2 inhibitors as furafylline and fluvoxamine (*K*_*i*_ = 0.12 − 3 μM, depending on a substrate), as well as in the range of the therapeutic concentrations of the neuroleptic tested [3, 4, 8, 9, 16]. Similarly, the *K*_*i*_ value for CYP2D6 inhibition by asenapine is close to the *K*_*i*_ values observed for such well-known inhibitors as tricyclic antidepressants or selective serotonin reuptake inhibitors, SSRIs [25]. The IC_50_ values obtained in our experiment confirm the ability of asenapine to strongly inhibit the activity of CYP1A2 and CYP2D6.

Although the therapeutic plasma concentrations of asenapine reach up to 0.1 μM [9, 26], its concentration in the liver may be several times higher than in the plasma owing to its physicochemical properties (log *P* = 4.9 for the neutral and 1.4 for cationic form; pK_a_ = 9.64 for amine and 8.6 for the protonated base) and related tissue distribution pattern [27, 28]. Basic lipophilic drugs are characterized by extensive accumulation in tissues due to nonspecific binding to cellular membranes and uptake by acidic subcellular compartments such as lysosomes [29–31]. These properties were shown in animal experiments for antidepressant drugs and phenothiazine neuroleptics in vitro [31–36] and in vivo [37, 38]. Moreover, it was observed in clinical studies that the concentration of thioridazine and its metabolites in the liver was 3- to 20-fold higher than that in the blood [39], while the concentration of haloperidol in the liver was 900-fold higher than that in plasma [40]. Hence, a potent inhibition of CYP1A2 and CYP2D6 by asenapine observed in vitro in the present study may be expected in vivo, since the K_i_ values calculated for human liver microsomes (3.2 and 1.75 μM, respectively) are close to the presumed concentration range for asenapine in the liver of psychiatric patients. In line with these findings, it was shown that asenapine doubled the concentration of paroxetine (a CYP2D6 substrate) in the blood plasma of psychiatric patients [12]. Since asenapine is both a substrate and an inhibitor of CYP1A2 and CYP2D6, it seems possible that this neuroleptic can also inhibit its own metabolism.

On the other hand, the inhibition of CYP3A4 by asenapine demonstrated in vitro in the present study seems to be of limited significance in vivo, since the calculated *K*_*i*_ values (27 − 31 μM) are above the presumed concentration range of asenapine in human liver during pharmacotherapy and the IC_50_ values are much higher than those obtained for CYP1A2 or CYP2D6.

The knowledge of the ability of asenapine to inhibit CYP1A2 and CYP2D6 is of pharmacological and clinical importance, since this drug is administered to patients for months or years, and very often in combination with other clinically important drugs that are substrates of the above-mentioned CYP enzymes. Therefore, the inhibition of those CYPs by asenapine may produce drug–drug interactions. The obtained results suggest that pharmacokinetic interactions involving asenapine and substrates of CYP2D6 (e.g., tricyclic antidepressants, SSRIs, codeine, dextromethorphan, debrisoquine, metoprolol, and propranolol) or CYP1A2 (e.g., caffeine, theophylline, phenacetin, tricyclic antidepressants, and propranolol) are likely to occur in patients during co-administration of the above-mentioned drugs. It is also worth stressing that caffeine, which was used in our experiment as a CYP1A2 marker substance, is also a component of numerous drugs and beverages, like coffee, tea, and energy drinks. Its therapeutic concentrations of 10–100 μM may be reached during high coffee consumption [41]. Considering a very broad spectrum of caffeine action on neuronal transmission [42] and the presented ability of asenapine to inhibit CYP1A2, a special caution should be taken when the two substances are administered jointly.

The obtained results may also have physiological and toxicological significance, since CYP1A2 and CYP2D6 are implicated in the biotransformation of endogenous substances, such as estradiol (CYP1A2) or neurosteroids (CYP2D6) and environmental contaminants polycyclic aromatic hydrocarbons (CYP1A2) [5, 43].

## Abbreviations

CYP: Cytochrome P450
HPLC: High-performance liquid chromatography
SSRIs: Selective serotonin reuptake inhibitors
